# Modes of Antiviral Action of Chemical Portions and Constituents from Woad Root Extract against Influenza Virus A FM1

**DOI:** 10.1155/2016/2537294

**Published:** 2016-02-18

**Authors:** Jia-Hang Su, Rui-Gang Diao, Shu-Guang Lv, Xiao-Dong Mou, Kefeng Li

**Affiliations:** ^1^Clinical Pharmacy Chamber, Yantai Traditional Chinese Medicine Hospital, Yantai, Shandong 264001, China; ^2^Clinical Pharmacy Chamber, Yantai Yuhuangding Hospital, Yantai, Shandong 264000, China; ^3^Heart Disease Division, Yantai Traditional Chinese Medicine Hospital, Shandong 264001, China; ^4^Department of Laboratory Medicine, Yantai Yuhuangding Hospital, Yantai, Shandong 264000, China; ^5^School of Medicine, University of California, San Diego, CA 92103, USA

## Abstract

Woad root has been used for the prevention of influenza for hundreds of years in many Asian countries. In this study, the antiviral modes of clemastanin B (CB), epigoitrin, phenylpropanoid portion (PEP), and the mixture of phenylpropanoids, alkaloids, and organic acid portions (PEP + ALK + OA) from wood root extract against influenza virus A FM1 were investigated. The results revealed that CB, epigoitrin, PEP, and PEP + ALK + OA exert their anti-influenza activity via inhibiting the virus multiplication, prophylaxis, and blocking the virus attachment. The primary mode of action of PEP and PEP + ALK + OA is the inhibition of virus replication. The inhibitory effect on virus attachment and multiplication is the main modes for epigoitrin. All the compounds or chemical portions from woad root extract tested in this study do not have direct virucidal activity. Our results provided the comprehensive analysis of the antiviral mechanism of wood root extract.

## 1. Introduction

Influenza or flu is one of the most significant acute respiratory diseases caused by the infection of influenza virus. Seasonal influenza affects millions of people in the world every year, leading to a serious threat to public health especially to children and the elderly. In addition, influenza virus has the potency to cause a severe pandemic and economic loss [1]. The outbreak of avian influenza A in China in 2013 caused nearly $6.5 billion in losses to the economy.

Currently, the synthetic antiviral drugs or vaccines have limited use in developing countries due to the emergence of resistant strains, the high cost, and the harmful side effects [2, 3]. However, anti-influenza agents derived from herbs have many advantages such as low cost and toxicity, extensive source, and ease of access [4, 5]. Moreover, herbal drugs usually have multitarget effects, which not only act as antiviral agents but also stimulate immunity [6]. Therefore, medicinal plant extracts and phytochemicals are attracting more and more attention as the potential sources for the development of new antiviral drugs during the recent decade.

Woad root (common name: Ban Lan Gen) is the dry root of plant* Isatis indigotica* Fort. Woad root was first documented as the herbal drug in* The Divine Husbandman's Herbal Foundation Canon*, a famous ancient medical book in the Han Dynasty of China (200 AD). It has been used in the treatment of cold, sore throat, and headache for hundreds of years in China [7, 8]. Woad root was used for the prevention of severe acute respiratory syndrome (SARS) in 2003 and swine flu pandemic in 2009 in China, Hong Kong, Taiwan, and Japan [9, 10].

Flu vaccines are the main prophylactic treatment in winter to protect against the influenza viruses. It was estimated that more than $3.2 billion was spent on flu vaccines production every year by the federal government of USA [11]. Therefore, it is necessary to develop the inexpensive drugs with effective prophylactic activity. The use of water extract of woad root to prevent cold has been documented for hundreds of years.

Recently, the antiviral effect of the methanol, water, and ethyl ester extract of woad root was confirmed through* in vitro* test [10, 12]. However, the modes of antiviral actions of these extract are still not clear. Additionally, there is little information on the differences of antiviral action between the single pure compound and the extract where the compound is isolated from.

**Figure 1:**
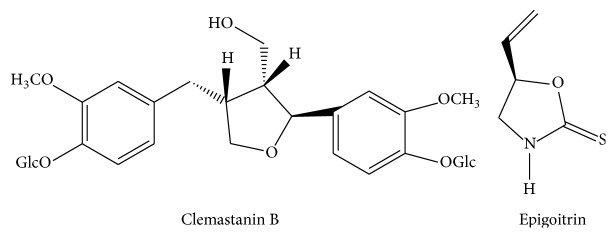
Chemical structure of clemastanin B (CB) and epigoitrin.

Phenylpropanoids (PEP), alkaloids, and organic acids are three major chemical portions in woad root. Clemastanin B (CB) is the most abundant compound which belongs to phenylpropanoid. Epigoitrin is the main alkaloid compound isolated from the woad root (Figure 1) [13, 14]. A previous screening showed that CB and epigoitrin have the strong inhibitory effect on influenza A1 virus FM1 [15]. The objective of the present study was to elucidate the possible anti-influenza mechanisms of CB and epigoitrin and compare with the phenylpropanoids portion and the mixture of phenylpropanoids, alkaloids, and organic acid portions (PEP + ALK + OA).

## 2. Materials and Methods

### 2.1. Viral Strains, Cell Lines, and Reagents

Mouse lung-adapted variant of influenza virus A FM1 strain was obtained from the Department of Microbiology and Immunology at Shandong University. The virus was propagated twice in the allantoic cavity of 9- to 10-day-old embryonated hen's eggs at 35°C for 48 h to enhance the virulence. The allantoic fluid was harvested for the measurement of its hemagglutinating activity. Once the hemagglutination titer reached 1 : 640, the virus was aliquoted and stored at −80°C until use. The Madin-Darby canine kidney (MDCK) cells and human cervical cancer (HeLa) cells were obtained from Institute of Cell Biology, Chinese Academy of Sciences. The positive control ribavirin (Batch number: 101018) was purchased from Baili Pharmaceutical Co. Ltd. in Sichuan province, China.

### 2.2. Preparation of Plant Extracts

The woad root was collected from Anhui province, China. Herb identification was confirmed through morphological and microscopic analysis according to the Chinese Pharmacopeia [16]. The extraction of pure compounds and chemical portions of woad root was conducted by Tianjin SunnyPeak Biotech. Co. Ltd. The mixture of phenylpropanoids, alkaloids, and organic acid portions (PEP + ALK + OA) was prepared by mixing each of the above portions at a ratio of 1 : 2 : 2 (w/w/w) [17]. The lyophilized materials were directly resuspended in the cell culture medium and filter sterilized through the 0.22 *μ*m membrane. For those compounds which cannot be dissolved in the medium, they were first dissolved in DMSO and then diluted with the fresh medium. The final DMSO concentration in the medium was less than 1%. The concentration of CB and epigoitrin was 50 *μ*g/mL and the initial concentration of PEP and PEP + ALK + OA was 100 *μ*g/mL.

### 2.3. Modes of Anti-Influenza Action

The anti-influenza action of CB, epigoitrin, and chemical portions from woad root extract was investigated in four different modes: therapeutic action, prophylaxis, direct virus inactivation, and inhibition of virus attachment.

### 2.4. Preincubation with Virus (Therapeutic Action of the Drugs)

The cells were preinfected with the virus before the pure compounds or chemical portions of plant extract were added. The therapeutic action of the drugs was evaluated by both cytopathic effects (CPE) reduction assay and cell MTT assay.

The CPE reduction assay was conducted according to the previous report with slight modifications [18]. Briefly, quadruplicate MDCK monolayer cells in 96-well plates were infected with 0.1 mL suspension containing 100 TCID_50_ (50% Tissue Culture Infective Dose) of virus for 2 h. The unabsorbed virus was then washed off using PBS. Quadruplicate cell monolayers were subsequently overlaid with 0.1 mL medium containing different nontoxic twofold serial dilutions of pure compounds, chemical portions of woad root extract. Cells with virus infection without drug treatment and the cells without virus and drugs were used as controls. The plates were incubated at 37°C under 5% CO_2_ for 72 h. The virus-induced CPE was observed under a light microscope in comparison with the parallel virus control and cell control.

The MTT reduction assay was performed according to the standard protocol [19]. In short, the experimental setup was the same with the procedures in CPE assay. After 3 days of incubation, 20 *μ*L of MTT was added to each well and incubated at 37°C for 4 h. Subsequently, DMSO was added and the absorbance was measured at 570 nm. The cells protection rate (%) was calculated by the following formula:

(1) $$\begin{array}{c} \left[\;\frac{{\text{OD}}_{\exp}-{\text{OD}}_{\text{virus control}}}{{\text{OD}}_{\text{cell control}}-{\text{OD}}_{\text{virus control}}}\;\right]\times 100\%. \end{array}$$

See [16].

### 2.5. Pretreatment with Drugs (Prophylaxis)

To evaluate the effects of pure compounds and chemical portions from woad extract on prophylaxis of cell infection, the MDCK monolayer cells in 96-well plates were overlaid with different nontoxic twofold serial dilutions of pure compounds, chemical portions of woad root extract. Four replicates were set up for each treatment and control. After 4 h, the test substances were removed from the wells and the monolayer cells were then infected with 100 TCID_50_ of influenza virus A FM1 at 37°C for 2 h to allow virus absorption. Subsequently, the unabsorbed virus was washed off using PBS and the equal amount of maintenance medium was added into each well. The plates were incubated at 37°C under 5% CO_2_ for 72 h. The virus-induced CPE was observed under light microscope.

### 2.6. Direct Virucidal Assay

The direct virucidal activity of the pure compounds and chemical portions from woad extract was tested according to the methods described by Carlucci et al. [20]. One hundred microliters of 100 TCID_50_ of the virus was treated with equal volumes of twofold diluted pure compounds or extract portions for 2 h at 37°C. The samples were then tenfold serially diluted. When the confluent monolayer of MDCK cells was formed, the surviving virus in the mixtures was determined in CPE assay and titers (TCID_50_ values) were calculated according to the Reed-Muench method.

### 2.7. Inhibition of Virus Attachment Assay

The monolayer of MDCK cells was cultured in 96-well plates. One hundred microliters of different nontoxic twofold serial dilutions of pure compounds, chemical portions of woad root extract, and the equal volume of 100 TCID_50_ of the virus were simultaneously added to MDCK cells [21]. After incubation of 2 h at 37°C, the virus/extract mixture was removed from the wells after which maintenance medium was added. The plates were incubated at 37°C under 5% CO_2_ for 72 h. The virus-induced CPE was observed under light microscope.

### 2.8. Statistical Analysis

All the experiments were repeated three times, each with quintuplicate determinations. The data were expressed as mean ± SD. Student's *t*-test was performed to compare between the control and treatments. A value of *p* < 0.05 was considered as significant difference (*∗*) and *p* < 0.01 was considered very significant (*∗∗*).

## 3. Results and Discussion

### 3.1. Therapeutic Action of the Pure Compounds and Chemical Portions from Woad Root Extract

**Figure 2:**
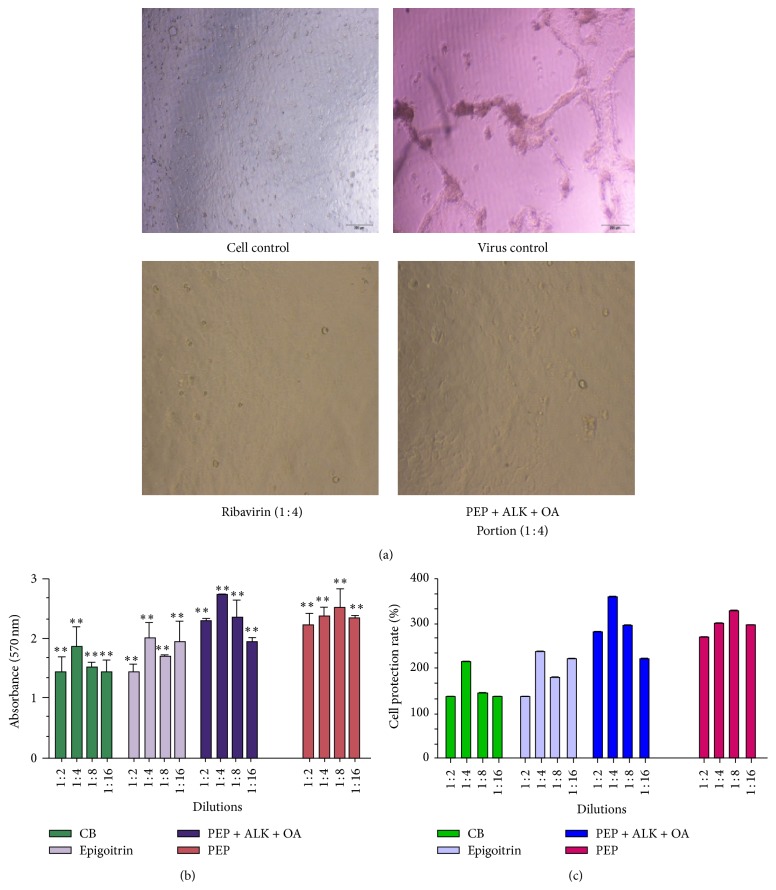
Effect of different dilutions of pure compounds and chemical portions from woad root on the viability of MDCK cells preinfected with 100 TCID_50_ of influenza A H1N1 virus: (a) microscopic analysis; (b) MTT assay; (c) cell protection rate (%). CB: clemastanin B; PEP + ALK + OA: the mixtures of phenylpropanoids, alkaloids, and organic acid portions. Cell control: normal MDCK cells without virus infection and drugs treatment; virus control: cells infected with the virus without drug treatments. Ribavirin (1 : 4): error bars represent standard deviation. The asterisks indicate a significant difference between the test samples and the virus control according to Student's *t*-test.

In order to investigate the therapeutic effect on influenza virus A, the cells were preinfected with the virus for 2 h followed by the addition of the antiviral compounds and chemical portions from woad root extract. The solvent blank, CB, epigoitrin, and chemical portions of woad root extract had no obvious cytotoxicity (data was not shown). The therapeutic action was evaluated by both CPE assay and MTT assay. Clear cytopathic effects were observed in MDCK cells infected with FM1 after 72 hours such as increased gaps between cells, rupture of the cell nucleus, and the partial or complete collapse of cells (Figure 2(a), virus control). In virus control group, 50% −75% of CPE was observed (see Table S1 in Supplementary Material available online at http://dx.doi.org/10.1155/2016/2537294). However, MDCK cells grow well in the drug treatment groups (Figure 2(a), ribavirin, and PEP + ALK + OA portion groups) and CPE formation was completely inhibited in all the dilutions (Table S1).

MTT reduction assay showed that the addition of CB, epigoitrin, PEP, and PEP + ALK + OA portions from woad root extract significantly increased the viability of MDCK cells preinfected with the virus compared with the virus control group in all the dilutions (*p* < 0.01) (Figure 2(b)). The molecular mechanisms of clemastanin B and epigoitrin for their antiviral activities have not been fully understood. A recent report showed that clemastanin B might target on viral endocytosis and retain the ribonucleoprotein (RNP) of the influenza virus in the nucleus [22]. Interestingly, the protection rate in four treatment groups was significantly higher than that in positive control ribavirin group under the same dilution (*p* < 0.05). This indicated that compounds and extract portions from woad root have better therapeutic action against influenza A virus FM1 than the current commercial synthetic antiviral drug ribavirin. Additionally, the protective effect of CB, epigoitrin, PEP, and PEP + ALK + OA portions was not dose-dependent. The highest protection rate was observed in 1 : 4 dilution of CB, epigoitrin, or the mixture of PEP + ALK + OA portions, while PEP diluted 1 : 8 resulted in the highest cell viability (Figure 2(c)). In comparison to different treatment groups, the mixture of PEP + ALK + OA portions (1 : 4) has the highest cell protection rate.

### 3.2. Prophylactic Action of the Pure Compounds and Chemical Portions from Woad Root Extract

In order to evaluate the effects of pure compounds and chemical portions from woad root extract on prophylaxis of influenza A virus, the MDCK cells were treated with serial dilution of CB, epigoitrin, PEP, and PEP + ALK + OA portions, respectively. After 4 h, the drugs were removed from the wells and the cells were infected with 100 TCID_50_. The CPE formation was observed and the cell viability was measured by MTT assay. CPE assay showed that there was no obvious CPE formation in MDCK cells pretreated with CB, epigoitrin, PEP, and PEP + ALK + OA portions in all the dilutions (Table S2).

As shown in Table 1, pretreatment with either pure compounds or chemical portions of woad root extract in all the dilutions significantly improved the viability of MDCK cells (*p* < 0.01). Moreover, compared with ribavirin, natural compounds or extracts from the wood root have higher prophylactic activity against influenza virus A FM1 (*p* < 0.01). The cell viability was dose-dependently increased by PEP and PEP + ALK + OA portions (Table 1). In contrast, the protection rate and cell viability were not significantly changed by the dilution of CB and epigoitrin (from 1 : 2 to 1 : 16). Among the four different natural products used in this study, PEP portion showed the most significant protective effect with the cell protection rate of 263.467%.

### 3.3. Direct Virucidal Action of the Pure Compounds and Chemical Portions from Woad Root Extract

**Figure 3:**
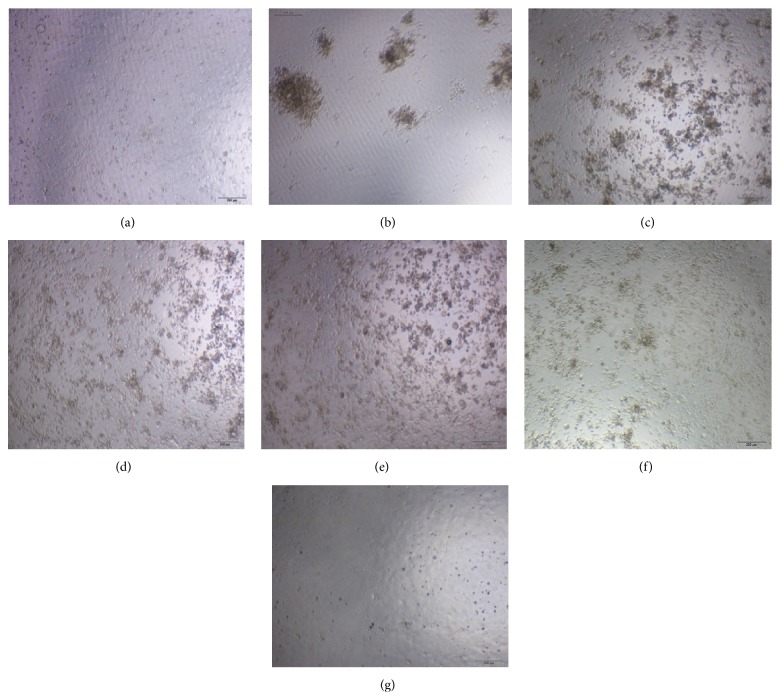
Direct virucidal effect of pure compounds and chemical portions from woad root extract on influenza A1 virus FM1: (a) cell control; (b) virus control; (c) CB (1 : 2); (d) epigoitrin; (e) PEP (1 : 2); (f) PEP + ALK + OA (1 : 2); (g) ribavirin (1 : 4).

Next, we investigated the effect of CB, epigoitrin, PEP, and PEP + ALK + OA portions on the inactivation of influenza virus A FM1 at 37°C. The cells without treatment were shown in Figure 3(a) and the virus control was in Figure 3(b). The MDCK cells infected with influenza virus and cocultured with CB, epigoitrin, PEP, or PEP + ALK + OA showed pyknosis condensation and even lysis (Figures 3(c)–3(f)). The surviving virus titer was 0.01 TCID_50_ in all the experimental groups. However, positive control ribavirin inhibits the CPE formation completely in all the dilution levels (Figure 3(g)). These observations revealed that CB, epigoitrin, PEP, and PEP + ALK + OA could not directly inactivate influenza virus A FM1 even at the concentration of 1 : 2 dilution. In a previous study, Hsuan et al. also found that the inhibition of pseudorabies virus by the methanol extract of woad leaves extract was not due to the direct virus inactivation [12].

### 3.4. Inhibitory Activity of the Pure Compounds and Chemical Portions from Woad Root Extract on Influenza Virus A FM1 Attachment

Influenza A virus attachment is primarily mediated by two types of glycoproteins called hemagglutinin and neuraminidase [23]. In this study, we investigated whether natural occurring compounds and chemical portions from woad extract inhibit virus attachment to the host cells. The results of CPE assay were listed in Table S3. There was no CPE formation in all the drug treatment groups even at the lowest concentration (1 : 16 dilution). The results suggested that CB, epigoitrin, PEP, and PEP + ALK + OA portions have a strong inhibitory effect on binding of influenza A virus to MDCK cells.

The viability of MDCK cells at 72 h after the infection of virus and simultaneous treatment with natural compounds was determined by MTT reduction assay. As shown in Table 2, the viability of MDCK cells was significantly increased as the result of drug treatments compared with virus control (*p* < 0.01). The inhibition of virus adsorption to the host cells by CB, epigoitrin, and PEP was in a dose-dependent manner. The maximum inhibitory effect of CB, epigoitrin, and PEP was observed with a 1 : 2 dilution, which resulted in 3.53, 3.99, and 4.43 times' increase in cell viability, respectively. In contrast, the highest cell protection rate in PEP + ALK + OA and positive control ribavirin groups was found in a 1 : 4 dilution. Additionally, it was shown that CB, epigoitrin, PEP, and PEP + ALK + OA are more effective than ribavirin on the inhibition of influenza A FM1 virus attachment (Table 2). When comparing four natural compounds and chemical portions, it was observed that PEP portion diluted 1 : 2 possessed the maximum inhibitory effect of virus absorption, which led to the highest protection of 273.218%.

**Figure 4:**
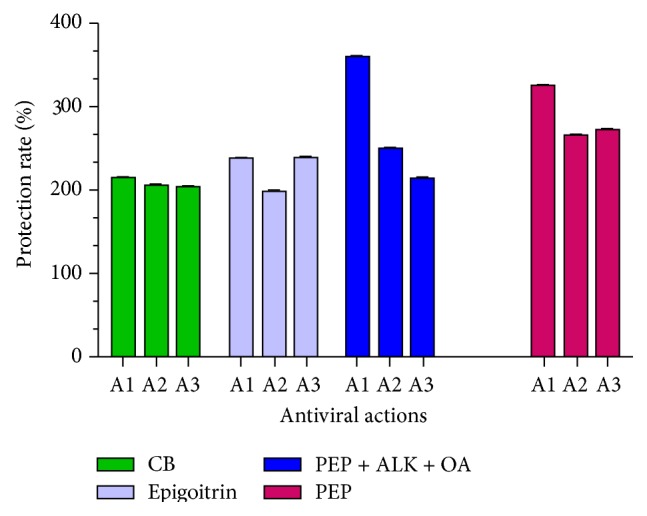
Differences on the major modes of antiviral action in the pure compounds and chemical portions from woad root extract. A1: therapeutic action; A2: prophylaxis; A3: inhibition of virus attachment. CB: clemastanin B; PEP + ALK + OA: the mixtures of phenylpropanoids, alkaloids, and organic acid portions. The concentration for CB: A1 (1 : 4 dilution), A2 (1 : 8 dilution), and A3 (1 : 2 dilution); epigoitrin concentration: A1 (1 : 4), A2 (1 : 16), and A3 (1 : 2); PEP + ALK + OA concentration: A1 (1 : 4), A2 (1 : 2), and A3 (1 : 4); PEP concentration: A1 (1 : 8), A2 (1 : 2), and A3 (1 : 2).

The classically defined antiviral mechanisms for medicinal plants include inhibiting virus replication, blocking virus attachment, direct inactivating the virus, and preventing from virus infection [24]. In this study, it was clearly demonstrated that CB, epigoitrin, PEP, or PEP + ALK + OA showed the anti-influenza activities by therapeutic action (inhibition of virus multiplication), prophylaxis, and inhibition of virus attachment. However, differences were observed on the major modes of antiviral action in different compounds and chemical portions (Figure 4). For instance, the highest cell protection rate in PEP or PEP + ALK + OA was from its therapeutic action (Figure 4, PEP, A1; PEP + ALK + OA, A1). The main anti-influenza modes for epigoitrin are the inhibition of virus multiplication and virus attachment (Figure 4, epigoitrin A1, epigoitrin A3). In contrast, three modes of antiviral action of CB contribute equally on the cell protection rate.

CB is the major phenylpropanoid compound in woad root and epigoitrin is the abundant alkaloid and indicator for the quality control of woad root [16]. Previous studies reported that the overall virus inhibitory effect of green tea is stronger in the plant total extract than the single pure compound from the extract due to the possible synergistic interactions between the ingredients in the extract [18, 25]. However, our results suggested that the change of antiviral activity might be because of the differences of antiviral mechanisms between the single compound and the mixture of the extract.

## 4. Conclusion

In the present study, the modes of anti-influenza action of the chemical portions and constituents from woad root extract were investigated. Our results revealed that CB, epigoitrin, PEP, or PEP + ALK + OA demonstrated their anti-influenza activities by therapeutic action, prophylaxis of cells, and inhibition of virus attachment. All the compounds or chemical portions tested do not have direct virucidal activity. The main antiviral mode for PEP and PEP + ALK + OA is the therapeutic action, while epigoitrin mainly inhibits the virus multiplication and attachment. To our knowledge, this is the first report on the antiviral mechanism of the compounds and chemical portions from woad root extract.

## Supplementary Material

Three tables (Table S1-S3) in the supplementary materials describe the formation of CPE (Cytopathic effect) in MDCK cells after the treatment of woad root extract. The cells were infected with pre-infected with Influenza Virus A FM1. Cells were then treated with the chemical portions and pure compounds from woad root extract in three different modes including therapeutic action, prophylaxis and direct virus inactivation.

## Figures and Tables

**Table 1:** Effect of prophylactic treatment on the viability and protection rate of MDCK cells.

Groups	OD_560_				Protection rate (%)			
	1 : 2	1 : 4	1 : 8	1 : 16	1 : 2	1 : 4	1 : 8	1 : 16
CB (50 *μ*g/mL)	2.126 ± 0.034^*∗∗*^	2.228 ± 0.167^*∗∗*^	2.284 ± 0.017^*∗∗*^	2.218 ± 0.011^*∗∗*^	188.348	199.08	205.023	198.08
Epigoitrin (50 *μ*g/mL)	2.197 ± 0.152^*∗∗*^	2.239 ± 0.050^*∗∗*^	2.202 ± 0.102^*∗∗*^	2.041 ± 0.036^*∗∗*^	195.871	200.29	196.344	214.435
PEP portion (1 mg/mL)	2.840 ± 0.150^*∗∗*^	2.464 ± 0.024^*∗∗*^	2.407 ± 0.048^*∗∗*^	2.149 ± 0.069 ^*∗∗*^	263.467	223.91	217.912	190.821
PEP + ALK + OA (1 mg/mL)	2.698 ± 0.082^*∗∗*^	2.090 ± 0.042^*∗∗*^	2.045 ± 0.089^*∗∗*^	2.171 ± 0.028^*∗∗*^	248.58	184.61	179.879	193.135
Ribavirin (100 *μ*g/mL)	1.866 ± 0.251^*∗∗*^	1.924 ± 0.306^*∗∗*^	1.614 ± 0.086^*∗∗*^	1.869 ± 0.016^*∗∗*^	161.047	167.1	134.245	161.31

Virus control		0.335 ± 0.073						
Cell control		1.286 ± 0.277						

PEP: phenylpropanoids portion; PEP + ALK + OA: the mixtures of phenylpropanoids, alkaloids, and organic acid portions. Cell control: normal cells without virus infection and drug treatments. Virus control: cells infected with influenza A FM1 virus. Data of OD_560_ was mean ± SD. The asterisks indicate a significant difference between the test samples and the virus control according to Student's *t*-test. ^*∗∗*^ *p* < 0.01.

**Table 2:** Inhibitory effect of pure compounds and chemical portions from woad root extract on influenza A FM1 virus attachment.

Groups	OD_560_				Protection rate (%)			
	1 : 2	1 : 4	1 : 8	1 : 16	1 : 2	1 : 4	1 : 8	1 : 16
CB (50 *μ*g/mL)	1.940 ± 0.262^*∗∗*^	1.665 ± 0.058^*∗∗*^	1.599 ± 0.161^*∗∗*^	1.368 ± 0.210^*∗∗*^	201.267	161.455	151.973	118.458
Epigoitrin (50 *μ*g/mL)	2.192 ± 0.108^*∗∗*^	1.980 ± 0.246^*∗∗*^	1.833 ± 0.049^*∗∗*^	1.639 ± 0.347^*∗∗*^	237.749	207.058	185.849	157.763
PEP portion (1 mg/mL)	2.437 ± 0.190^*∗∗*^	2.340 ± 0.123^*∗∗*^	2.020 ± 0.319^*∗∗*^	1.695 ± 0.041^*∗∗*^	273.218	259.175	212.921	165.798
PEP + ALK + OA (1 mg/mL)	2.043 ± 0.072^*∗∗*^	2.055 ± 0.009^*∗∗*^	1.743 ± 0.282^*∗∗*^	1.599 ± 0.069^*∗∗*^	216.178	217.988	172.819	151.900
Ribavirin (100 *μ*g/mL)	1.614 ± 0.093^*∗∗*^	1.736 ± 0.307^*∗∗*^	1.441 ± 0.001^*∗∗*^	1.279 ± 0.018^*∗∗*^	154.144	171.734	129.099	105.646

Virus control		0.549 ± 0.690						
Cell control		1.24 ± 0.675						

PEP: phenylpropanoids portion; PEP + ALK + OA: the mixtures of phenylpropanoids, alkaloids, and organic acid portions. Cell control: normal cells without virus infection and drug treatments. Virus control: cells infected with influenza A FM1 virus. Data of OD_560 _was mean ± SD. The asterisks indicate a significant difference between the test samples and the virus control according to Student's *t*-test. ^*∗∗*^ *p* < 0.01.
