# Expression of Concern on “Modes of Antiviral Action of Chemical Portions and Constituents from Woad Root Extract against Influenza Virus A FM1”

**DOI:** 10.1155/2018/5810294

**Published:** 2018-05-30

**Authors:** Evidence-Based Complementary and Alternative Medicine

*Evidence-Based Complementary and Alternative Medicine* would like to express our concern with the article titled “Modes of Antiviral Action of Chemical Portions and Constituents from Woad Root Extract against Influenza Virus A FM1” published in* Evidence-Based Complementary and Alternative Medicine* in January 2016 [1]. The article presents the same results as another article, titled “Antiviral activities against influenza virus (FM1) of bioactive fractions and representative compounds extracted from Banlangen (Radix Isatidis)” and published in the* Journal of Traditional Chinese Medicine* in June 2016 by different authors [2]. The* Journal of Traditional Chinese Medicine* article was accepted in August 2015, before the* Evidence-Based Complementary and Alternative Medicine* article was submitted in November 2015.

Around half of the text is the same; Figure 1 is identical; Figure 2(a) in* Evidence-Based Complementary and Alternative Medicine* is the same as Figures 2(c)–2(f) in the* Journal of Traditional Chinese Medicine*; Figure 2(b) shows the same results, though presented differently, as Figures 2(a) and 2(b); Figure 3 is identical, except that panel (b) is rotated 180° and panel (f) is different; Table 1 is identical to Table 3; Table 2 is identical to Table 5; Figure 4 shows the same results; all but one of the references also appear in the* Journal of Traditional Chinese Medicine* reference list. Although one article uses the term “woad root” and the other “Banlangen* (Radix Isatidis),*” these are the same plant.

The* Evidence-Based Complementary and Alternative Medicine* authors said that the work comes from Jian-Hang Su's M.S. thesis and is not the same as that published in the* Journal of Traditional Chinese Medicine*; their article focuses on the root extract, while the* Journal of Traditional Chinese Medicine* article focuses on the dose response and pure compounds. The* Journal of Traditional Chinese Medicine* authors said that the work was from Weiyang Ye's 2011 Ph.D. thesis. Both groups of authors presented evidence to support their positions.

We asked the institutions of both groups of authors to investigate. The University of California, San Diego, found an investigation was not within its remit and we did not receive a response from the other institutions, Yantai Traditional Chinese Medicine Hospital, Yantai Yuhuangding Hospital, and Nanjing University of Chinese Medicine. Because the outcome of our internal investigation and the institutional investigations has been inconclusive, the journal will not retract the article at this time. However, readers should be aware of the duplication of these results.
