# Supplementary material for: Modes of Antiviral Action of Chemical Portions and Constituents from Woad Root Extract against Influenza Virus A FM1
Source: Evid Based Complement Alternat Med. 2016 Feb 18;2016:2537294. doi: 10.1155/2016/2537294 (PMC4775799; doi:10.1155/2016/2537294)
Supplement: Supplementary file 1 — Three tables (Table S1-S3) in the supplementary materials describe the formation of CPE (Cytopathic effect) in MDCK cells after the treatment of woad root extract. The cells were infected with pre-infected with Influenza Virus A FM1. Cells were then treated with the chemical portions and pure compounds from woad root extract in three different modes including therapeutic action, prophylaxis and direct virus inactivation. [file 2537294.f1.pdf]

Supplemental Tables

Table S1 ~~The~~ Formation of CPE in MDCK cells pre-infected with influenza A H1N1 virus

Groups	Initial concentration	IC50	Dilutions			
			1:2	1:4	1:8	1:16
CB	50 µg/ml	1.5 µg/ml	–	–	–	–
Epigoitrin	50 µg/ml	1.3 µg/ml	–	–	–	–
Phenylpropanoids portion	1 mg/ml	0.02 mg/ml	–	–	–	–
Phenylpropanoids+alkaloids+organic acid portions	1 mg/ml	0.015 mg/ml	–	–	–	–
Ribavirin	100 µg/ml	2.5 µg/ml	–	–	–	–
Cell control			–	–	–	–
Virus Control			++±	++±	++±	++±

The CPE (Cytopathic effect) was graded as follows: "-"= 0% CPE; "+"=0%-25% CPE; "++"=26%-50% CPE; "+++"=51%-75% CPE; "++++"=76%-100%. Cell control: Normal cells without virus infection and drug treatments. Virus control: Cells infected with influenza A FM1 virus.

Table S2 ~~The~~ Formation of CPE in MDCK cells pre-treated with pure compounds and chemical portions of woad root extract

Groups	Initial concentration	IC50	Dilutions			
			1:2	1:4	1:8	1:16
CB	50 µg/ml	14 µg/ml	–	–	–	–
Epigoitrin	50 µg/ml	14.5 µg/ml	–	–	–	–
Phenylpropanoids portion	1mg/ml	0.2 mg/ml	–	–	–	–
Phenylpropanoids+alkaloids+organic acid portions	1mg/ml	0.25 mg/ml	–	–	–	–
Ribavirin	100 µg/ml	36.5 µg/ml	–	–	–	–
Cell control			–	–	–	–
Virus Control			+++	+++	+++	+++

The CPE was graded as follows: "-"= 0% CPE; "+"=0%-25% CPE; "++"=26%-50% CPE; "+++"=51%-75% CPE; "++++"=76%-100%. Cell control: Normal cells without virus infection and drug treatments. Virus control: Cells infected with influenza A FM1 virus.

Table S3 ~~The~~ Formation of CPE in MDCK cells induced by virus attachment

Groups	Initial concentration	IC50	Dilutions			
			1:2	1:4	1:8	1:16
CB	50 µg/ml	28.3 µg/ml	–	–	–	–
Epigoitrin	50 µg/ml	25.1 µg/ml	–	–	–	–
Phenylpropanoids portion	1mg/ml	0.45 mg/ml	–	–	–	–
Phenylpropanoids+alkaloids+organic acid portions	1mg/ml	0.55 mg/ml	–	–	–	–
Ribavirin	100 µg/ml	47.8 µg/ml	–	–	–	–
Cell control			–	–	–	–
Virus Control			+++	+++	+++	+++

The CPE was graded as follows: "-"= 0% CPE; "+"=0%-25% CPE; "++"=26%-50% CPE; "+++"=51%-75% CPE; "++++"=76%-100%. Cell control: Normal cells without virus infection and drug treatments. Virus control: Cells infected with influenza A FM1 virus.
